# Availability and accessibility of services to address financial toxicity described by Australian lung cancer patients and healthcare professionals

**DOI:** 10.1007/s00520-023-08019-4

**Published:** 2023-09-02

**Authors:** Holly Chung, Amelia Hyatt, Suzanne Kosmider, Kate Webber, Meinir Krishnasamy

**Affiliations:** 1 Academic Nursing Unit, Peter MacCallum Cancer Centre, 305 Grattan Street Parkville, Melbourne, Victoria 3000 Australia; 2 Department of Health Services Research, Peter MacCallum Cancer Centre, 305 Grattan Street Parkville, Melbourne, Victoria 3000 Australia; 3 Sir Peter MacCallum Department of Oncology, University of Melbourne, Melbourne, Victoria 3010 Australia; 4 Department of Nursing, University of Melbourne, Melbourne, Victoria 3010 Australia; 5 Cancer Services, Sunshine Hospital, Western Health, St Albans, Victoria 3021 Australia; 6 Oncology Department, Monash Health, Clayton, Victoria 3168 Australia; 7 School of Clinical Sciences, Monash University, Clayton, Victoria 3800 Australia; 8 Victorian Comprehensive Cancer Centre Alliance, Melbourne, Victoria 3000 Australia

**Keywords:** Financial toxicity, Lung cancer, Patient experience, Qualitative, Cancer supportive care, Oncology

## Abstract

**Purpose:**

Although the financial burden and impact of a cancer diagnosis has been widely described in international literature, less understood is the availability and accessibility of services to ameliorate this need. This study reports the experiences of Australian lung cancer patients and health professionals delivering care, regarding factors that exacerbate and mitigate financial stress, and availability and accessibility of services to support people following a cancer diagnosis.

**Methods:**

Qualitative semi-structured interviews with twenty-three lung cancer patients attending two metropolitan tertiary health services and eleven health professionals delivering care were undertaken during July–August 2021.

**Results:**

Neither health service systematically screened for financial toxicity nor routinely provided information regarding potential financial impacts during consultations. Patients experienced lengthy delays in accessing welfare supports, provoking financial stress and worry. Health professionals reported limited resources and referral services to support patients with financial need; this was especially problematic for patients with lung cancer. They described its psychological impact on patients and their family members or carers and warned of its impact on ability to adhere to treatment.

**Conclusion:**

Available and accessibility of services addressing financial toxicity in Australian lung cancer patients is inadequate. Although financial stress is a common, distressing problem, health professionals feel hampered in their ability to help due to limited service availability. Left unaddressed, financial toxicity can impact treatment adherence, directly influencing health outcomes, and increase risk of poverty, amplifying social inequities. Findings highlight opportunity for actionable interventions like financial consent and routine screening and discussion of financial toxicity across care pathways.

## Introduction

Universal health coverage (UHC) is defined as access to health services for all people as they need them, when and where they need them, without financial hardship [1]. Financial toxicity (the financial burden and impact of out-of-pocket (OOP) costs, loss of income or savings) associated with a cancer diagnosis can have severe consequences, ranging from debt or bankruptcy to impacting people’s ability to adhere to or complete treatment, impacting survival [2, 3]. Despite international commitment to achieving UHC [1], financial toxicity remains a significant issue linked to poor cancer outcomes [4], particularly for those already disadvantaged by the impact of social determinants of health [5].

Australian health service coverage (comprising two tiers of health systems, ‘public’ and ‘private’) is regarded favourably in pursuing UHC compared to other high-income countries [6]. Nevertheless, rising OOP contributions for patients accessing cancer treatment and care across both public and private health systems are increasingly resulting in financial toxicity [7, 8]. Particularly vulnerable to the impact of OOP costs are those who identify as female, younger, culturally diverse, unemployed, or live far from treatment centres [9–11]. Additionally, specific cancer diagnoses, such as lung cancer, are associated with higher risk of toxicity, predominantly due to social determinants of health [12, 13]. Current national and international cancer policies overtly focus on reducing disparities in cancer incidence, experiences, and outcomes [14, 15]. Addressing socioeconomic disadvantage and financial toxicity resulting from cancer will be key to achieving equitable cancer outcomes.

Literature regarding experiences of financial toxicity among people affected by cancer, has informed clinical (service) level interventions such as informed financial consent, financial counselling, and de-implementation of low-value treatments as opportunities to prevent or mitigate financial stress in cancer patients [16, 17]. In Australia, there is a lack of evidence to demonstrate how effective these interventions are in terms of addressing the key drivers of financial toxicity. Additionally, the extent to which service-level interventions to address financial toxicity are available (implemented into routine care) and accessible (ease of access to existing services) is unclear [18]. Therefore, this study investigated lung cancer patients and healthcare professionals’ experiences regarding availability and accessibility of services to address financial toxicity and describe its consequences when left unmanaged.

## Methods

### Approach

A qualitative exploratory study was undertaken using semi-structured interviews with lung cancer patients and health professionals involved their care. This paper reports a subset of data from a larger study on cancer supportive care needs among people affected by lung cancer [19], funded by the Victorian Government Department of Health Cancer Support, Treatment and Research Unit and approved by the Peter MacCallum Cancer Centre Human Research Ethics Committee (multi-site approval number: HREC/66771/PMCC).

### Setting and sampling

Patient and health professional participants were recruited from two metropolitan tertiary health services (health services A & B). The services were similar in that they are busy and growing cancer services, have large multicultural patient cohorts, serve disadvantaged communities, and have limited specialist cancer nursing/care co-ordination capability to undertake comprehensive supportive care needs screening. People with a recently confirmed lung cancer diagnosis (> 3 months and < 2.5 years when screened for eligibility to take part), and able to participate in a qualitative interview in English, were recruited. A purposive recruitment strategy was used to include participants with a range of experiences, including age, sex, and cultural background. Health professionals included multidisciplinary clinicians and health service managers involved with the planning and delivery of lung cancer services.

### Data collection

Patient participants were identified from clinic lists with the assistance of authors SK and KW and invited to participate by phone due to COVID-19 restrictions between June and August 2021. Trained qualitative researchers conducted interviews with participants over telephone or video-conferencing depending on participant preference. Written informed consent was obtained and verbal consent audio-recorded prior to interviews.

Semi-structured interview guides comprised a subset of question prompts specific to financial toxicity. These explored patient-identified priority concerns and services most helpful in supporting or managing financial toxicity and barriers to accessing support. Health professional interviews comprised prompts investigating services available, their accessibility and consequences for patients, those of most importance, and perceptions of health service ability to address financial toxicity concerns.

Patient demographic and clinical characteristics recorded at interview commencement included the following: age, sex, marital and employment status, Aboriginal or Torres Strait Islander identification, first language, highest level of education attained, time since cancer diagnosis, and type/stage of cancer if known. Health professional and service manager characteristics recorded were as follows: sex, role, and time employed at health service. Recruitment ended when data saturation was achieved (when data from additional interviews reiterated content generated in previous interviews) [20]. All interviews were audio-recorded and transcribed verbatim.

### Analysis

Demographic (quantitative) characteristics of participants were analysed descriptively. Qualitative transcripts were analysed in NVivo 12 (QSR International) using Interpretive Description, a methodology developed to inform clinical practice improvements [21]. A coding frame was developed to explicitly explore experiences regarding financial toxicity, services available to address this need, and associated outcomes.

## Results

### Participants

Thirty-four participants took part in qualitative interviews: 23 patients (68%) and 11 health professionals (32%). Participant demographics are presented in Table 1 below.

**Table 1:** Participant demographic characteristics at time of interview

	Health service A	Health service B	Total
*Patients*	**(*n*** **= 15)**	**(*n*** **= 8)**	**(*n*** **= 23)**
Age (years)			
Mean, SD	67, 8	67, 10	67, 8
Min, Max	57, 82	53, 77	53, 82
Time since diagnosis (months)*			
Mean, SD	11, 7	15, 7	12, 7
Min, Max	5, 31	4, 24	4, 31
Gender	***n*** (%)**	***n*** (%)**	***n*** (%)**
Male	6 (40)	4 (50)	10 (43)
Female	9 (60)	4 (50)	13 (57)
English first language			
Yes	9 (60)	4 (50)	13 (57)
No	6 (40)	4 (50)	10 (43)
Marital status			
Single	2 (13)	2 (25)	4 (17)
Married/de facto	6 (40)	4 (50)	10 (43)
Separated/divorced	6 (40)	1 (13)	7 (30)
Widowed	1 (7)	1 (13)	2 (9)
Aboriginal and/or Torres Strait Islander			
No	15 (100)	8 (100)	23 (100)
Current employment status			
Employed (full time/part time)	1 (7)	0 (0)	1 (4)
Not in paid employment	6 (40)	4 (50)	10 (43)
Taking sick or personal leave			
Retired	8 (53)	4 (50)	12 (53)
Highest level of education			
Partial secondary	10 (67)	7 (88)	17 (74)
Completed secondary (year 12)	2 (13)	0 (0)	2 (9)
Trade/TAFE	1 (7)	1 (13)	2 (9)
University	2 (13)	0 (0)	2 (9)
Diagnosis			
Lung cancer (did not specify)	10 (67)	5 (62)	12 (52)
NSCLC stage III/IV	5 (33)	3 (38)	11 (47)
Healthcare professionals	**(*n*** **= 5)**	**(*n*** **= 6)**	**(*n*** **= 11)**
Time at healthcare service (years)			
Mean, SD	16, 11	12, 5	15, 10
Min, Max	6, 37	8, 20	6, 37
Gender	***n*** (%)**	***n*** (%)**	***n*** (%)**
Male	2 (40)	2 (33)	4 (36)
Female	3 (60)	4 (67)	7 (64)
Role category			
Doctor	1 (20)	4 (67)	5 (46)
Nurse	3 (60)	1 (17)	4 (36)
Allied health	1 (20)	1 (17)	2 (18)
Stakeholder			
Healthcare professional	4 (80)	4 (67)	8 (73)
Healthcare service manager	1 (20)	2 (33)	(27)

*N = 22

**Percentages may not total 100 due to rounding

### Qualitative analysis

**Fig. 1:**
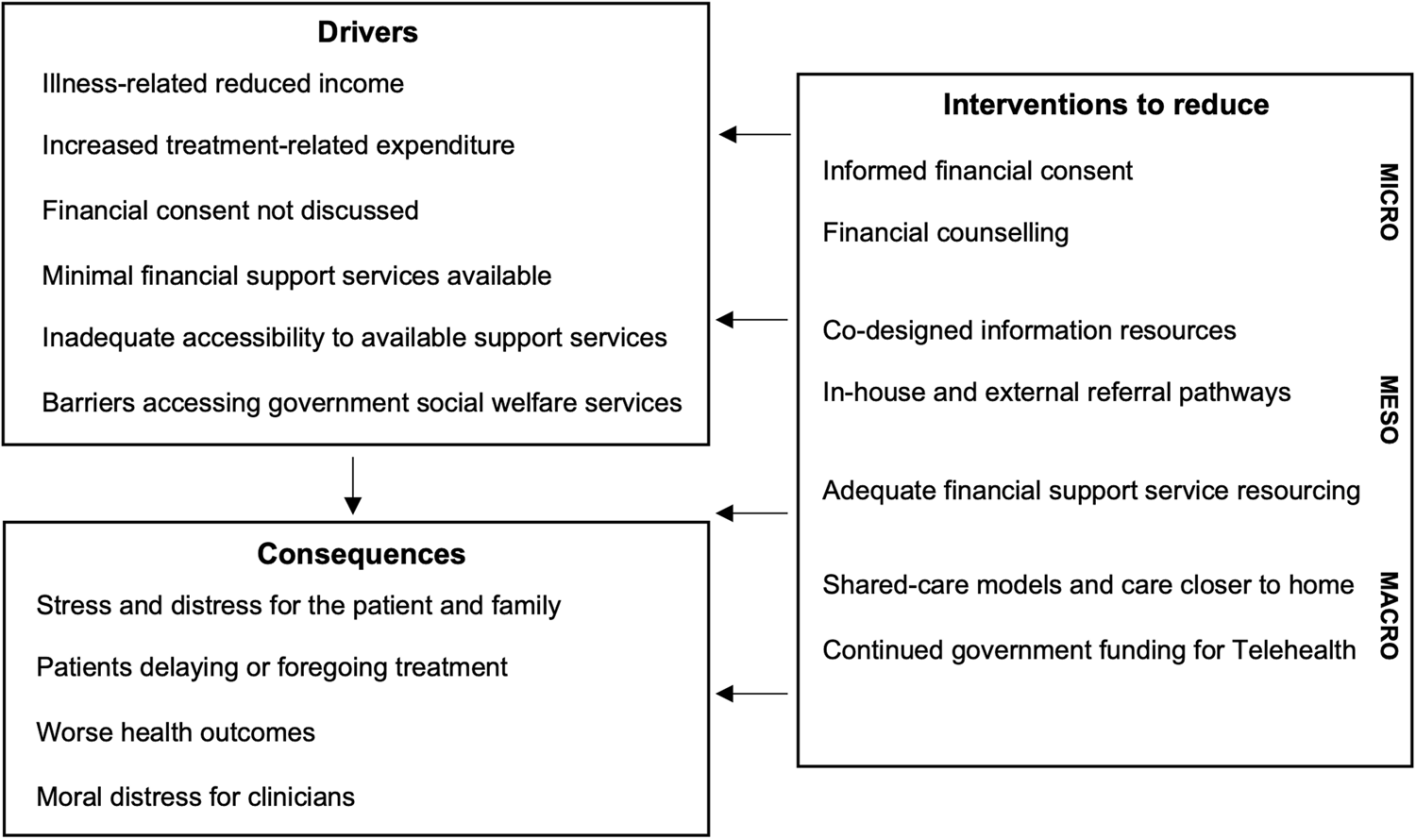
Drivers, consequences, and interventions to address financial toxicity

Qualitative analysis investigated participant experiences and perceptions regarding availability and accessibility of services to address financial toxicity. Four themes were developed: “screening and communication about financial toxicity”, “referrals to, and availability of, in-house, community, and non-profit organisation services”, “challenges with government social welfare services”, and “consequences of unmanaged financial toxicity”. Drivers and consequences described are presented in Fig. 1.

### Availability and accessibility of services to address financial toxicity

#### Screening and communication about financial toxicity

Most health professionals at both health services stated that they did not routinely discuss financial concerns with patients, and no patient participants reported discussing financial costs of treatment with their clinical team.

If they don't get seen by social work, I don't think anybody actually asks about the financial impacts of their treatment. - HPB4

The cost factor wasn’t mentioned at all, wasn’t mentioned at all. – PB1

Lack of information about the range of factors likely to result in, or exacerbate financial toxicity, meant that patients did not recognise it as a concern they could share with their treating team. This influenced their knowledge of, or motivation to pursue, social welfare benefits.

Maybe finances are a mess, bills haven’t been paid, and maybe money’s really tight because family didn’t know that they could get carers payments, and so someone’s had to take unpaid leave from work to be a carer. -HPB5

To access services, patients had to advocate for assistance. But health professionals cited lower health literacy, language barriers, and financial insecurity prior to diagnosis as barriers asking for help or assistance with financial issues.

If they are a lot more health literate then they understand usually to ask for extra help if they need it. Whereas it’s those ones that either don’t have enough of a health understanding or they don’t speak the language…lack of support and things that really need those services put in place, they’re the ones that are not getting screened. – HPB4

Despite a lack of communication about financial toxicity in practice across both health services, health professionals emphasized the importance of navigating patients to financial counselling and social welfare as soon as possible after diagnosis, to prevent financial toxicity and the distress associated with it.

People need different support, carers allowances, can the carer take time off, driving. So, it’s more about practical help and finances. – HPB5

Ongoing expenses meant financial issues quickly escalated if not identified early, resulting in heightened stress for patients and families which, according to health professionals, could result in a worsening of symptoms or inability to adhere to treatment:

They either get worse side effects or, you know, they get labelled as non-compliant, they don’t turn up to appointments because it’s too hard. – HPB4

#### Referrals to, and availability of, in-house, community, and non-profit organisation services

Health professionals reported limited ability to refer patients, particularly those attending out-patient clinics, to relevant professional service-level support (e.g. social work). Accessing out-patient support services was described as particularly problematic due to funding models in Australia that split health funding between Federal (outpatient) and State (inpatient) governments. Moreover, even when referrals were progressed, staffing constraints within each health service prevented availability of timely consultations.

Consequently, many health professionals felt that the only support they could provide patients for financial toxicity was information regarding social welfare and referrals to non-government organisations (NGOs). Health professionals discussed the lack of NGO and community support services for patients with lung cancer, particularly compared with those available for other cancer groups, such as breast or prostate cancer.

One of the challenges is the availability of community supports that are specific to lung cancer patients. You sort of get a whole suite for other cancers. The first one that comes to mind really is breast cancer; so, there’s lots and lots of not-for-profit community support available for the breast cancer patients. But we find that with lung cancer patients we don’t have as many resources available to us in the community to refer onto. – HPB8

#### Challenges with government social welfare services

Being “linked in” to or having previous experience with welfare services such as disability support or age pensions prior to diagnosis was helpful for patients. This minimised delays to accessing benefits and meant that people were already familiar with navigating services. Several patients reported leveraging other circumstances to help mediate financial toxicity, such as using the “Covid-19 Early Access” program to access superannuation (Australia’s defined contribution retirement pension fund). Although this helped address immediate concerns, it generated concerns about future financial security.

If they then need to access things like Centrelink payments it takes time so, you know, that’s a period of no financial support for them that could … the ball could’ve started rolling right from the start. -HPB8

At the moment I’m okay because I got a super payout but other than that if I didn’t get that I’d probably financially be in trouble – PB10

I don’t have a great deal of money. Like, I don’t know how long I’m gonna live for, it might last me for five years and that’s being very frugal. – PA20

#### Consequences of unmanaged financial toxicity

For most patients, current access to services provided by government welfare systems was described as slow, complex, and confusing. Difficulty with easily and quickly accessing welfare supports resulted in patients choosing to delay treatment or missing appointments due to prioritising employment to keep financially afloat. One health professional described a patient who had to choose between receiving treatment or providing for his family. These delays compromised health outcomes or resulted in patients presenting to hospital acutely unwell.

I’ve had to learn to cope with a pension. There’s no spare money. – PA14

We’ve had one patient who is the primary carer for his two kids and he’s actually not turned up for several treatments because he’s had to look after the kids or he’s had to actually work to provide for the kids. So it impacts our ability to provide adequate care… – HPA2

Clinicians involved in delivery of cancer treatments described trying to help patients complete paperwork to access welfare service but struggled to do so because of the time pressure in busy clinical settings. Time spent completing paperwork to address financial concerns while trying to focus on treatment issues was described as challenging and was compounded by a lack of resources to refer patients to. Knowing that delays in addressing financial concerns might impact people’s ability to access treatment and care resulted in an emotional toll on health professionals.

When people… have these financial issues they’re highly stressed, they’re frustrated, they’re angry, and they have to take it out on social workers or nurses or people around them. So, yeah, that impacts everybody as well. - HPA5

…Also on an emotional level, it does affect the nurses too.

HPA2

## Discussion

While experiences of financial toxicity are well described from the perspective of cancer patients and health professionals in studies from comparable, publicly funded health systems (e.g. Canada and the UK) [2, 22, 23], our study contributes new insight, illustrating system-level challenges that impact availability of and access to services to redress financial toxicity among Australians affected by lung cancer. Our data lend support to evidence that financial toxicity is an avoidable consequence of cancer and its treatment that disproportionately affects those most disadvantaged. Our study also advances knowledge in this field by proposing actionable interventions to ameliorate this priority concern, shown in Fig. 1.

Firstly, there is a need to better inform and prepare patients for the potential of financial toxicity. This finding is consistent with Australian and international evidence, and recent Australian cancer policies for Informed Financial Consent which advocates for comprehensive definition and improved information at diagnosis regarding cumulative effects of financial toxicity [24–26]. Early discussion of financial stresses should be integrated as standard of care, along with timely access to services, supports, and financial counselling to mitigate toxicity, particularly for those experiencing financial stress at diagnosis [27]. Improved definition of both objective and subjective components of financial toxicity may improve both capacity educate patients, as well as understand (therefore address) the prevalence of this priority concern within and across cancer populations [28].

Secondly, health professionals were acutely aware of potentially devastating consequences of financial toxicity yet reported rarely discussing this issue during consultations, in line with a recent Australian survey [29]. Insufficient resources or access to services may explain why financial concerns were not routinely discussed as part of clinical consultations; these are consistently reported barriers in literature [17, 30]. Health professionals require clear pathways for referral to in-house or external services, and these services must be sufficiently resourced to meet population need. The moral distress experienced by health professionals supporting patients through difficult decisions regarding prioritising treatment needs over income presents an area for future investigation.

Thirdly, previous Australian policies have recognised the need to address and mitigate financial toxicity [31]; however, these policies have not translated into practice change. The new Australian Cancer Plan [14] offers an opportunity to target prevalent and distressing consequences of cancer; for example, by co-locating social welfare experts or financial counsellors within health services as a core component of the cancer care team to ensure timely access to available supports [11].

The fourth opportunity for intervention draws on findings from this study and others [7, 27] that demonstrate urgent need for revision of Australia’s Medicare system (responsible for the provision of universal health coverage) to tackle financial toxicity, ensuring that a cancer diagnosis does not send any Australian into, or exacerbate, poverty.

Reform of financial consent, literacy, and service capability to respond and to prevent unnecessary distress and sub-optimal and inequitable health outcomes presents a fifth composite target for action to mitigate generational poverty from cancer.

Interventions for financial toxicity need to enhance access to services and promote patient self-management. The COVID-19 pandemic showed telehealth to be a beneficial and acceptable to patients and health professionals, removing travel costs and the need to take time from work [32], which may be particularly useful for individuals living in regional and rural locations [33]. However, attention is required to those most at risk of financial stress and their capacity to invest in the technology required for optimal telehealth consultations [34]. Continued government investment to protect telehealth as a subsidised (Australian Medicare-billable item) is essential, while investment in shared-care models between general practitioners and specialist services, and expansion of models of care such as home-delivery of cancer therapies, offers practical and patient-endorsed solutions to hospital-based care. Ongoing investment in “care closer to home” supported by telehealth offers opportunity to minimise financial burden associated with travel for cancer treatment and care.

Finally, co-designed navigation resources (such as websites) that target what matters most to people with cancer can improve equity of access to information and services relevant to patients [35].

It is important to note that many of the drivers and consequences of financial toxicity described in this paper are not exclusive to the Australian context. Efforts by Carrera et al., at depicting the potential cascade of financial consequences following a cancer diagnosis in America, present opportunity to map timepoints where people may be at particular risk of financial toxicity [36]. Our study has highlighted actionable interventions which can be implemented and tested for efficacy in removing or reducing the impact of “financial cascade timepoints”.

Substantive and growing evidence demonstrates the prevalence and impact of financial toxicity experienced by people affected by cancer across different regions and health systems globally [23, 37, 38].

Urgently addressing financial toxicity at individual, health system, and government levels is crucial to achieving universal health coverage and equity in cancer care.

### Strengths and limitations

This study drew on the experiences of a small sample of people recently diagnosed with lung cancer and multidisciplinary health professionals in metropolitan health services in Australia, although almost half (43%) of our sample reported English as a second language. Not captured are the perspectives of people in regional/remote settings, First Nations peoples, or cancer survivors, who are known to experience greater risk of financial toxicity [7, 39]. Importantly, few of our participants were working at time of data collection, so specific challenges faced by working lung cancer patients are probably underrepresented. Study recruitment was impacted by COVID-19-related disruptions resulting in slower recruitment and lower participation rates that we had aimed for [40]. Despite this, our findings offer important insights into the experiences and consequences of financial toxicity and the scarcity of services available to address it. Exploring the experiences of individuals with lung cancer presents an important perspective on financial toxicity due to established links between lung cancer incidence and social disadvantage. Our work adopted a contemporary, gold-standard approach to equity research, focusing on service improvements for the most disadvantaged as a way of improving the health system for all [41]. We have demonstrated how availability of and access to service provision in our study is inadequate to achieve policy objectives regarding equitable cancer care, which is at odds with principles of universal health coverage. It is likely that these findings are applicable to other public health services and cancer patient groups in Australia. Transferability of findings to other health system contexts may be limited.

## Conclusion

Financial toxicity has a profound impact on patients with lung cancer. Our study demonstrates that financial toxicity is a mitigable side effect of cancer and its treatments, but services available to participants in this study, were insufficient to address peoples’ needs. Our data provide important evidence of the need for standardised and embedded discussion of the financial impact of a cancer diagnosis as standard of care, and greater investment into financial support services to help people affected by cancer identify their risk of, and manage, financial toxicity.

## Data Availability

The datasets generated during the current study are available from the corresponding author on reasonable request and HREC approval conditions.

## References

[1] World Health Organization (2022). Universal health coverage (UHC).

[2] Longo CJ, Fitch MI, Banfield L, Hanly P, Yabroff KR, Sharp L (2020). Financial toxicity associated with a cancer diagnosis in publicly funded healthcare countries: a systematic review. Support Care Cancer.

[3] Chan RJ, Gordon LG, Tan CJ, Chan A, Bradford NK, Yates P (2019). Relationships between financial toxicity and symptom burden in cancer survivors: a systematic review. J Pain Symptom Manag.

[4] Shah K, Zafar SY, Chino F (2022). Role of financial toxicity in perpetuating health disparities. Trends in Cancer.

[5] Abbott DE, Voils CL, Fisher DA, Greenberg CC, Safdar N (2017). Socioeconomic disparities, financial toxicity, and opportunities for enhanced system efficiencies for patients with cancer. J Surg Oncol.

[6] The World Bank. UHC service coverage index – High income, Australia Washington D.C2019 [cited 2023 05 May]. Available from: https://data.worldbank.org/indicator/SH.UHC.SRVS.CV.XD?locations=AU

[7] Bygrave A, Whittaker K, Paul C, Fradgley EA, Varlow M, Aranda S (2021). Australian experiences of out-of-pocket costs and financial burden following a cancer diagnosis: a systematic review. Int J Environ Res Public Health.

[8] Rodriguez-Acevedo AJ, Chan RJ, Olsen CM, Pandeya N, Whiteman DC, Gordon LG (2021). Out-of-pocket medical expenses compared across five years for patients with one of five common cancers in Australia. BMC Cancer.

[9] Yousuf ZS (2016). Financial toxicity of cancer care: it’s time to intervene. J Natl Cancer Inst.

[10] Tucker-Seeley RD, Yabroff KR (2015) Minimizing the “financial toxicity” associated with cancer care: advancing the research agenda. J National Cancer Inst 108(5):djv410. 10.1093/jnci/djv410 10.1093/jnci/djv410 26657336

[11] Lentz R, Benson AB, Kircher S (2019). Financial toxicity in cancer care: prevalence, causes, consequences, and reduction strategies. J Surg Oncol.

[12] Forrest LF, Adams J, Wareham H, Rubin G, White M (2013). Socioeconomic inequalities in lung cancer treatment: systematic review and meta-analysis. PLoS Med.

[13] Mitra D, Shaw A, Tjepkema M, Peters P (2015). Social determinants of lung cancer incidence in Canada: a 13-year prospective study. Health Rep.

[14] Cancer Australia (2022). Australian Cancer Plan Canberra.

[15] Dunn J (2023). It is time to close the gap in cancer care.

[16] Desai A, Gyawali B (2020) Financial toxicity of cancer treatment: Moving the discussion from acknowledgement of the problem to identifying solutions. EClinicalMedicine 20:100269 10.1016/j.eclinm.2020.100269 PMC7152810 32300733

[17] Abrams HR, Durbin S, Huang CX, Johnson SF, Nayak RK, Zahner GJ (2021). Financial toxicity in cancer care: origins, impact, and solutions. Transl Behav Med.

[18] Levesque JF, Harris MF, Russell G (2013). Patient-centred access to health care: conceptualising access at the interface of health systems and populations. Int J Equity Health.

[19] Hyatt A, Chung H, Aston R, Gough K, Krishnasamy M (2022). Social return on investment economic evaluation of supportive care for lung cancer patients in acute care settings in Australia. BMC Health Serv Res.

[20] Saunders B, Sim J, Kingstone T, Baker S, Waterfield J, Bartlam B (2018). Saturation in qualitative research: exploring its conceptualization and operationalization. Qual Quant.

[21] Thorne S (2016). Interpretive description: qualitative research for applied practice.

[22] Moffatt S, Noble E, Exley C (2010). " Done more for me in a fortnight than anybody done in all me life." How welfare rights advice can help people with cancer. BMC Health Serv Res.

[23] Fitch MI, Sharp L, Hanly P, Longo CJ (2022). Experiencing financial toxicity associated with cancer in publicly funded healthcare systems: a systematic review of qualitative studies. J Cancer Surviv.

[24] Breast Cancer Network Australia, Cancer Council Australia, CanTeen, Prostate Cancer Foundation of Australia. Standard for informed financial consent. Canberra: Cancer Australia; 2020. chrome-extension://efaidnbmnnnibpcajpcglclefindmkaj/https://www.cancer.org.au/assets/pdf/standard_for_informed_financial_consent

[25] Carlson MA, Fradgley EA, Bridge P, Taylor J, Morris S, Coutts E (2022). The dynamic relationship between cancer and employment-related financial toxicity: an in-depth qualitative study of 21 Australian cancer survivor experiences and preferences for support. Support Care Cancer.

[26] Yeager KA, Zahnd WE, Eberth JM, Vanderpool RC, Rohweder C, Teal R, Vu M, Stradtman L, Frost EL, Trapl E, Gonzalez SK, Vu T, Ko LK, Cole A, Farris PE, Shannon J, Askelson N, Seegmiller L, White A, Edward J, Davis M, Petermann V, Wheeler SB (2023). Financial navigation: Staff perspectives on patients' financial burden of cancer care. J Cancer Surv Res Prac.

[27] Longo CJ, Gordon LG, Nund RL, Hart NH, Teleni L, Thamm C (2022). Clinical management of financial toxicity–identifying opportunities through experiential insights of cancer survivors, caregivers, and social workers. Curr Oncol.

[28] Pauge S, Surmann B, Mehlis K, Zueger A, Richter L, Menold N (2021). Patient-reported financial distress in cancer: a systematic review of risk factors in universal healthcare systems. Cancers..

[29] Gordon LG, Nabukalu D, Chan RJ, Goldsbury DE, Hobbs K, Hunt L, Karikios DJ, Mackay G, Muir L, Leigh L, Thamm C, Lindsay D, Whittaker K, Varlow M, McLoone J, Financial Toxicity Working Group, O. B. O. T. C (2023). Opinions and strategies of Australian health professionals on tackling cancer-related financial toxicity: A nationwide survey. Asia-Pacific JCO.

[30] Ragavan M, Parikh D, Patel M (2021). Defining the clinician’s role in mitigating financial toxicity: an exploratory study. Support Care Cancer.

[31] Commonwealth of Australia, House of Representative. Speech Canberra: Hansard; 2019. p. 2011–6 Shorten, Bill, MP. https://www.aph.gov.au/parliamentary_business/hansard

[32] Donelan K, Barreto EA, Sossong S, Michael C, Estrada JJ, Cohen AB (2019). Patient and clinician experiences with telehealth for patient follow-up care. Am J Manag Care.

[33] Skrabal Ross X, Gunn KM, Olver I (2021). Understanding the strategies rural cancer patients and survivors use to manage financial toxicity and the broader implications on their lives. Support Care Cancer.

[34] Chang JE, Lai AY, Gupta A, Nguyen AM, Berry CA, Shelley DR (2021). Rapid transition to telehealth and the digital divide: implications for primary care access and equity in a post-COVID era. Milbank Q.

[35] State of Victoria, Department of Health and Human Services, September (2020) Victorian cancer plan 2020-2024. page 57. chrome-extension://efaidnbmnnnibpcajpcglclefindmkaj/https://www.health.vic.gov.au/sites/default/files/migrated/files/collections/research-and-reports/v/victorian-cancer-plan-2020-2024_improving-cancer-outcomes-for-all-victorians.pdf

[36] Carrera PM, Kantarjian HM, Blinder VS (2018). The financial burden and distress of patients with cancer: Understanding and stepping-up action on the financial toxicity of cancer treatment. CA Cancer J Clin.

[37] Eala MAB, Dee EC, Ginsburg O, Chua MLK, Bhoo-Pathy N (2022). Financial toxicities of cancer in low-and middle-income countries: perspectives from Southeast Asia. Cancer..

[38] Collado L, Brownell I (2019). The crippling financial toxicity of cancer in the United States. Cancer Biol Ther.

[39] Koczwara B (2017). Unemployment after cancer-a hidden driver of financial toxicity.

[40] Leong TL (2021). Delayed access to lung cancer screening and treatment during the COVID-19 pandemic: are we headed for a lung cancer pandemic?. Respirology..

[41] Winters N, Venkatapuram S, Geniets A, Wynne-Bannister E (2020). Prioritarian principles for digital health in low resource settings. J Med Ethics.

